# Study on Sulfide Stress Corrosion Cracking Susceptibility of 20# Steel Weld Joints in H_2_S-Rich Environments

**DOI:** 10.3390/molecules30234499

**Published:** 2025-11-21

**Authors:** Xinze Li, Yanqi Ran, Zhiming Yu, Ting Mao

**Affiliations:** 1 College of Engineering, China University of Petroleum (Beijing) at Karamay, Karamay Campus, Karamay 834000, China; lixinze@cupk.edu.cn (X.L.); 2023216901@st.cupk.edu.cn (Y.R.); 2 Research Institute of Natural Gas Technology, PetroChina Southwest Oil and Gas Field Company, Chengdu 610299, China; maoting01@petrochina.com.cn

**Keywords:** 20# steel, stress corrosion, hydrogen sulfide, stress corrosion cracking susceptibility

## Abstract

This study aims to clarify the influence of H_2_S concentration and temperature on the sulfide stress corrosion cracking (SSCC) susceptibility of 20# steel weld joints. 20# steel is a commonly selected pipe material for ground gas pipelines, and there is a risk of welds cracking after years of service. The selection of a corrosive environment is based on the working conditions of high-sulfur oil fields on site. Slow strain rate tensile (SSRT) tests were conducted under simulated high-sulfur gathering and transportation conditions across varying temperatures and H_2_S concentrations. The mechanical properties, SSCC susceptibility, fracture morphology, and elemental composition of fracture surfaces were systematically analyzed. As H_2_S concentration increased (5%, 7.5%, 10%) and temperature decreased (30–60 °C), the elongation after fracture and the reduction in area of 20# steel decreased, while the yield strength and SSCC susceptibility increased. The H_2_S concentration range of 0–5% represented a critical sensitivity interval for the material, where elongation after fracture decreased by up to 74%. Within the 5–10% H_2_S range, elongation decreased by only 2.11%, indicating a slowing trend of fracture toughness deterioration, though SSCC susceptibility still increased by 12%. Increasing the temperature from 30 °C to 60 °C reduced SSCC susceptibility by approximately 30%, confirming higher susceptibility at lower temperatures. Temperature exerts a lesser influence on material performance than H_2_S concentration. Also, 20# steel remains within the SSCC brittle fracture sensitivity zone in sulfur-containing environments, necessitating strict quality control to avoid defects and stress concentrations. In high-sulfur environments (H_2_S > 5%), further increases in H_2_S concentration have a diminished effect on cracking susceptibility.

## 1. Introduction

In the development of oil and gas fields, H_2_S and CO_2_, as associated gases produced during petroleum extraction, cause severe corrosion issues in pipeline materials. In high-sulfur oil and gas fields, the risk of sulfide stress corrosion cracking (SSCC) in pipelines is particularly prominent, with the primary corrosion types being uniform corrosion and stress corrosion cracking (SCC) [1,2,3]. Among these, stress corrosion cracking predominantly occurs in the girth weld zones, characterized by concealment and suddenness. The time to failure can range from a few hours to several years, often without obvious early warning signs. Conventional inspection methods struggle to detect microcracks in their initial stages. However, once cracks initiate, they can lead to sudden and catastrophic failure within a short period, posing extremely high risks [4,5].

In recent years, certain progress has been made in the study of stress corrosion behavior in pipeline steels. Xu et al. [6] found that the mechanical properties of corroded steel deteriorate with increasing corrosion severity, leading to ductility degradation. Liu et al. [7] observed that as the hydrogen sulfide (H_2_S) concentration rises, the stress corrosion susceptibility of steel increases while its corrosion-resistant lifespan decreases. Abdullah [8] analyzed the stress–strain curves of corroded steel through tensile tests and noted that while yield strength and ultimate tensile strength remained largely unchanged, elongation significantly decreased. Zhou et al. [9] investigated hydrogen diffusion behavior in low-carbon tubing steel under varying H_2_S partial pressures, concluding that H_2_S partial pressure alters the composition and structure of corrosion product films, thereby affecting hydrogen diffusion. Ikeda et al. [10] demonstrated that in CO_2_ environments with H_2_S partial pressures exceeding 0.003 MPa, steel is highly prone to sulfide stress corrosion cracking (SSCC). These findings indicate that H_2_S partial pressure significantly influences the corrosion behavior of metallic materials in high-pressure environments.

In wet H_2_S environments, the degradation of steel begins with electrochemical corrosion. Dissolved H_2_S facilitates the penetration of hydrogen atoms (H^+^) into the steel. Some of these hydrogen atoms accumulate at internal defects (such as inclusions and microcracks) and combine to form hydrogen molecules, creating significant internal pressure that leads to hydrogen blistering (HB). When these blisters connect to form step-shaped cracks, hydrogen-induced cracking (HIC) occurs, a process that requires no external stress. Simultaneously, hydrogen dissolved in the crystal lattice embrittles the steel. If tensile stress (external or residual) is present, sulfide stress corrosion cracking (SSCC) may initiate, often occurring in high-hardness areas such as near welds [11,12].

Stress corrosion cracking (SCC) results from the combined effects of stress and corrosive media, with its critical threshold dependent on material properties, environmental conditions, and applied stress. However, existing research on corrosion behavior lacks quantitative studies on the stress corrosion susceptibility of materials in H_2_S environments. The understanding of stress corrosion cracking sensitivity in environments with hydrogen sulfide content greater than 5% is not clear enough.

20# steel has a better balance of strength, plasticity, and weldability compared to other carbon steel materials. The proportion of ferrite and pearlite in 20# steel is relatively moderate [13]. This study focuses on aged 20# steel pipelines with girth welds that have been in service for nearly 30 years, examining their mechanical properties and stress corrosion behavior under varying H_2_S concentrations and temperatures in H_2_S/CO_2_ environments. This article quantitatively analyzes the effects of hydrogen sulfide content and temperature on material stress corrosion sensitivity in environments with hydrogen sulfide content greater than 5%.

## 2. Experimental Section

### 2.1. Test Materials

#### 2.1.1. Material Composition

**Figure 1:**
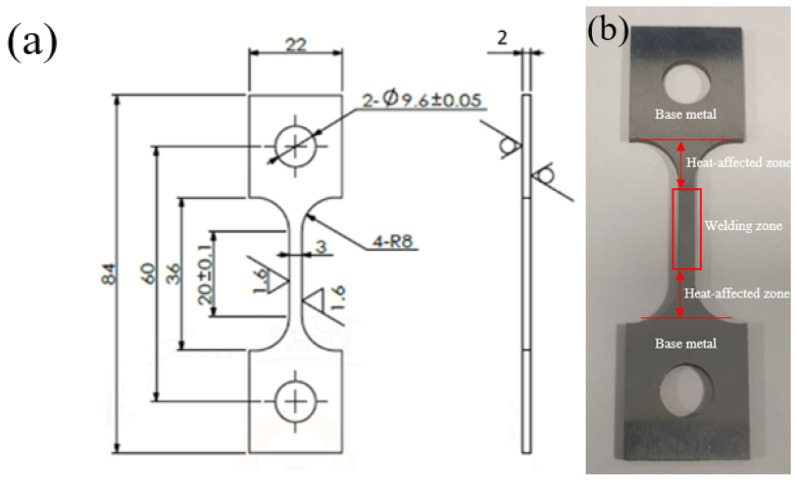
Sample parameter diagram: (**a**) 20# geometry and dimensions of steel specimens/mm; (**b**) sample physical image.

The material was selected from the old 20# steel on site, and the sample was prepared by wire cutting technology and processed into a plate-shaped sample. The sample is 84 mm long and 22 mm wide, with a small hole diameter of 10 mm and a wheelbase of 20 mm. The specific dimensions are shown in Figure 1a, and the actual sample is shown in Figure 1b. The sample is welded to the base metal using arc welding, and the welding rod model is E4303 of the GB5117-2012 “Covered electrodes for manual metal are welding ofnon-alloy and fine grain steels” [14] standard, which complies with the standard GB 50683-2011 “Code for Acceptance of Construction Quality of Field Equipment and Industrial Pipeline Welding Engineering” [15]. The welding area of the failed part of the pipeline was selected for chemical composition analysis. The results are shown in Table 1.

#### 2.1.2. Microstructure Analysis

**Figure 2:**
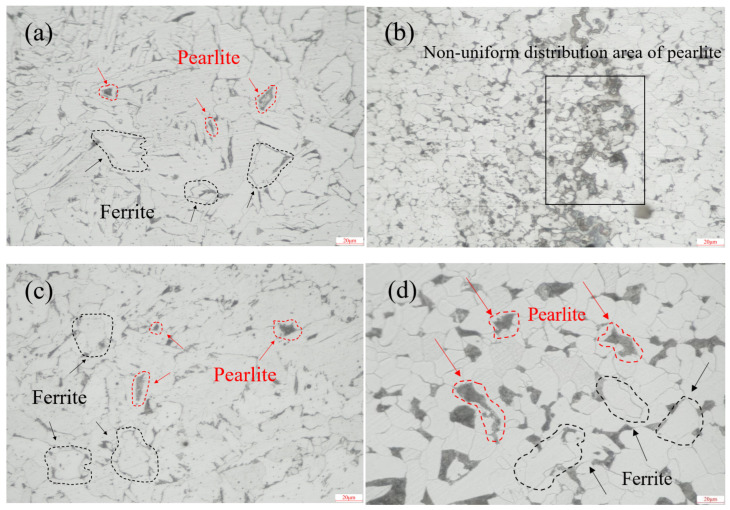
Metallographic structure diagram: (**a**) cap weld zone (500× magnification), (**b**) fill weld zone (500× magnification), (**c**) root weld zone (500× magnification), (**d**) base material (500× magnification).

Cut a 5 mm × 5 mm sample from the weld area and progressively grind the cross-section using 400 to 1200 grit metallographic sandpaper. Then, perform fine polishing with 2000-grit polishing paste on polishing paper. Etch the polished cross-section with a 2 wt% nitric acid alcohol solution and observe the metallographic structure using a metallographic microscope. Figure 2 shows the metallographic microstructure of the weld zone and base metal. As illustrated, the cap weld zone and root weld zone primarily consisted of ferrite (white structure) and pearlite (black structure) [16]. The fill weld zone exhibited non-uniform pearlite distribution. The base metal microstructure consisted of ferrite and pearlite with a uniform grain size.

**Figure 3:**
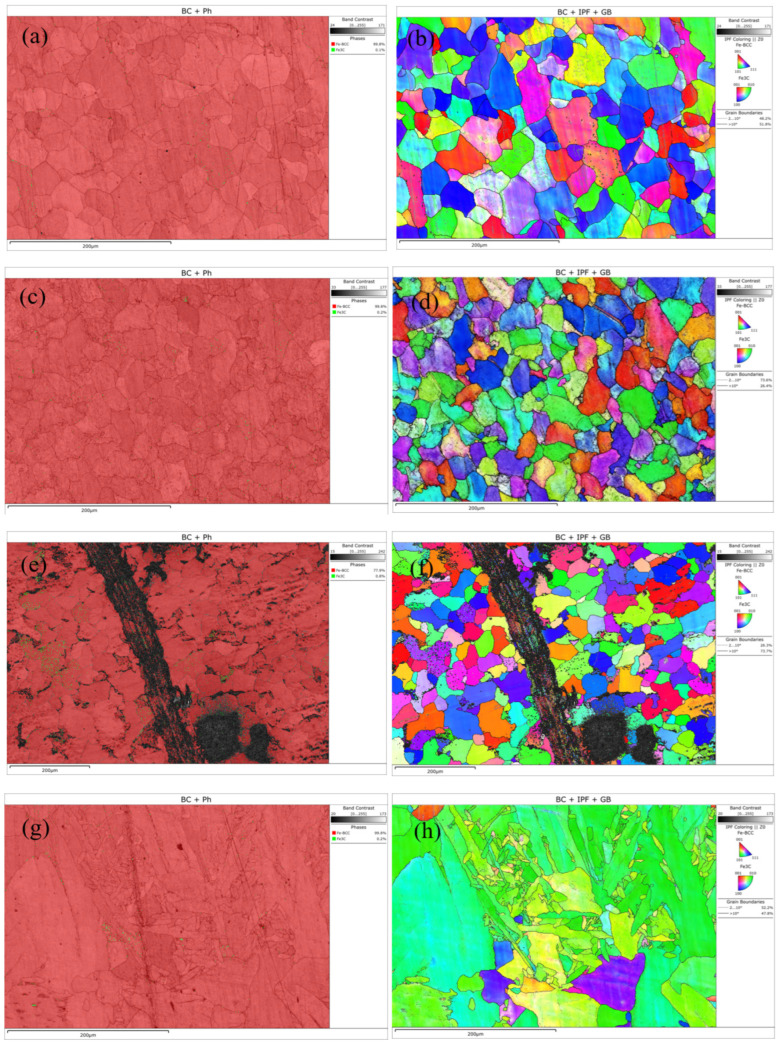
EBSD maps of different regions: (**a**,**b**) Base metal; (**c**,**d**) heat-affected zone; (**e**,**f**) cracks; (**g**,**h**) weld zone.

EBSD technology effectively reveals the evolution of microstructure and crystallographic characteristics in different regions of welded joints due to variations in thermal history and strain history. The equipment parameters were as follows: SEM model—Thermo Scientific Apreo ChemiSEM; EBSD detector—TruePix (Thermo Scientific, Waltham, MA, USA). The EBSD results are shown in Figure 3. The base metal exhibits a polychromatic grain orientation distribution, indicating an initial state of random texture without significant directional processing or heat treatment effects. In the IPF map of the heat-affected zone (HAZ), a large number of fine newly formed grains with colors distinctly different from the base metal can be observed, accompanied by a more complex color distribution. This suggests that the HAZ underwent intense recovery and recrystallization processes under the welding thermal cycle. In the IPF map around the crack tip and propagation path, extreme color gradients and sharp orientation changes can be observed, providing direct evidence of severe plastic deformation and intense lattice rotation. This reveals a high degree of local strain concentration, with cracks tending to propagate along regions of high strain gradients, where the path is closely related to microstructural mismatches. The IPF map of the weld metal displays a typical directionally solidified structure, characterized by coarse columnar grains. Within individual columnar grains, the uniform color indicates a single crystallographic orientation, while the stark color contrast between different columnar grains leads to significant anisotropy in the mechanical properties of the weld.

### 2.2. Mechanical Performance Analysis

According to the requirements of SY/T 0452-2021 “Standard for welding procedure qualification of oil and gas metal pipeline” [17] and GB/T 9711-2023 “Steel pipe for pipeline transportation systems” [18], the maximum allowable hardness value for the steel pipe base material and weld seam is 250HV10. The hardness values of the steel pipe base metal and weld are shown in Table 2, which comply with the requirements of the standard mentioned above. According to the standards GB/T 2653-2008 “Welding Street Bending Test Method” [19] and SY/T 0425-2021 “Welding Procedure Evaluation of Petroleum and Natural Gas Metal Pipelines” [20], bending tests were carried out on the welds of failed fittings. All specimens showed cracks, among which the specimen subjected to a back-bending test showed a crack with a length of 15.63 mm, indicating that the root welding part was relatively weak. The mechanical performance parameters of the base material are shown in Table 3.

### 2.3. Experimental Conditions and Methodology

We used sandpaper to grind and polish the samples. The experimental conditions included an air blank group (nitrogen-purged air) and simulated high-sulfur oilfield water. We calculated the pH values of the experimental conditions using the OLI Analyzer Studio (OLI Systems, Parsippany, NJ, USA); the specific results are shown in Table 4. Temperature conditions were set at 30 °C and 60 °C; CO_2_ concentration at 5%; H_2_S concentrations at 5%, 7.5%, and 10%; and pressure was maintained at 8 MPa. The simulation was carried out in the laboratory using a temperature control system and a gas transmission device. The tests conducted in air served as a control group for comparison. Field water quality monitoring results are shown in Table 5.

The temperature range of the on-site gathering and transmission pipeline was between 30 °C and 60 °C, and the pressure was around 8 MPa. The carbon dioxide content in the gathering pipeline was around 5%, and the hydrogen sulfide content greater than 5% was considered as high-sulfur conditions. The slow strain rate tensile test uses a high-temperature and high-pressure slow tensile testing machine, equipment model CFS-50, supplier Shanghai Cor-Force Stress-Corrosion Test Equipment Co, Ltd. (Shanghai, China), in accordance with GB/T15970.7-2017 [21], where specimens were tested at a strain rate of 1.0 × 10^−6^ s^−1^ under specified environmental conditions to evaluate mechanical properties, and the loading mode adopted constant rate loading; stress corrosion susceptibility indices (including elongation sensitivity factor F_δ_ and reduction in area sensitivity factor F_ε_) were determined through calculation with Formulas (1) and (2) for assessing the SCC susceptibility of 20# steel [22,23].

(1) $$F_{\delta }=(\delta_{a}-\delta_{c})/\delta_{a}\times 100\%$$

(2) $$F_{\varepsilon }=(\varepsilon_{a}-\varepsilon_{c})/\varepsilon_{a}\times 100\%$$

In the formula, $\delta_{a}$ represents the post-fracture elongation in air, $\delta_{c}$ denotes the post-fracture elongation in the test solution, $\varepsilon_{a}$ indicates the reduction in area in air, and $\varepsilon_{c}$ stands for the reduction in area in the test solution.

Higher $F_{\delta }$ and $F_{\varepsilon }$ values indicate greater stress corrosion susceptibility, making the material more prone to stress corrosion cracking (SCC) in the specified environment. The material is considered across the following situations: in a safe zone ($F_{\delta }$, $F_{\varepsilon }$ < 25%) with low SCC susceptibility, where cracking will not occur; in a critical zone (25% ≤ $F_{\delta }$, $F_{\varepsilon }$ < 35%) with potential SCC risk; or in a brittle fracture zone ($F_{\delta }$, $F_{\varepsilon }$ ≥ 35%), where SCC will inevitably occur [24,25,26].

Fracture surface morphology was characterized using a ZEISS EVO 18 SEM (Jena, Germany, 20 kV accelerating voltage, 27 mm working distance), while corrosion products were analyzed by an OXFORD X-Max EDS (Abingdon, UK) with a 20 mm^2^ detection area.

## 3. Results and Discussion

### 3.1. Effect of Hydrogen Sulfide

#### 3.1.1. Macro-Morphological Analysis

**Figure 4:**
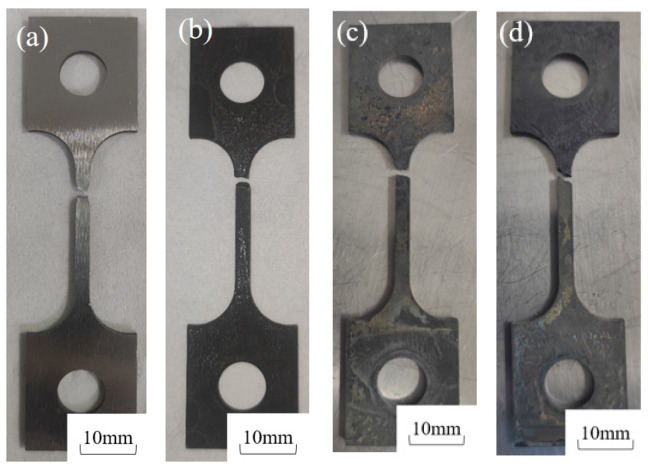
Macro-fracture morphology of 20# steel specimens after testing at 30 °C under different H_2_S concentrations: (**a**) 30 °C air, (**b**) 30 °C + 5% H_2_S + 5% CO_2_, (**c**) 30 °C + 7.5% H_2_S + 5% CO_2_, (**d**) 30 °C + 10% H_2_S + 5% CO_2_.

Figure 4 shows the post-fracture macro-morphology under different H_2_S concentrations (5%, 7.5%, 10%) at 30 °C. In air, the 20# steel exhibited deformation with evident necking at the fracture surface [27]. However, in H_2_S/CO_2_-containing environments, the specimen surface lost metallic luster, turning dull black due to corrosion, while the fracture surface became flat without necking. The fracture time in air was 69 h, whereas in H_2_S environments (5%, 7.5%, 10%), it drastically reduced to 17, 15, and 12 h, representing decreases of 75.4%, 78.2%, and 82.6%, respectively.

#### 3.1.2. Mechanical Properties

**Figure 5:**
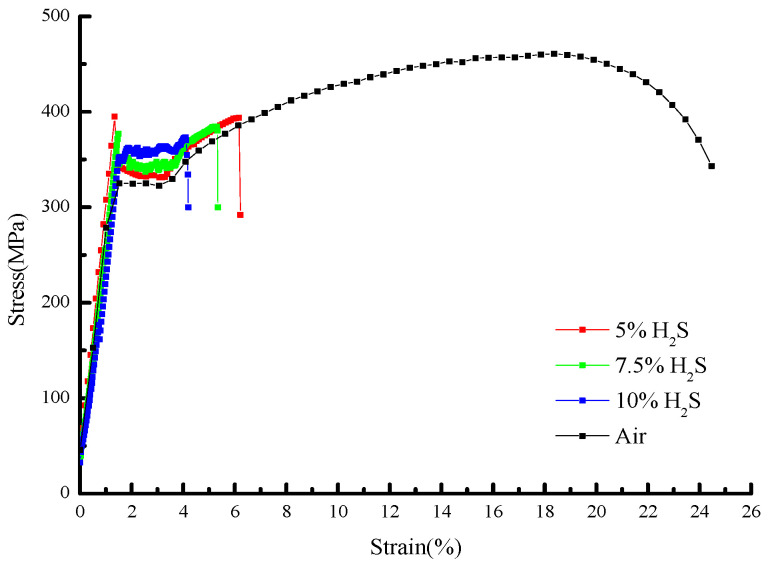
Stress–strain curves of 20# steel under different H_2_S concentrations at 30 °C.

**Figure 6:**
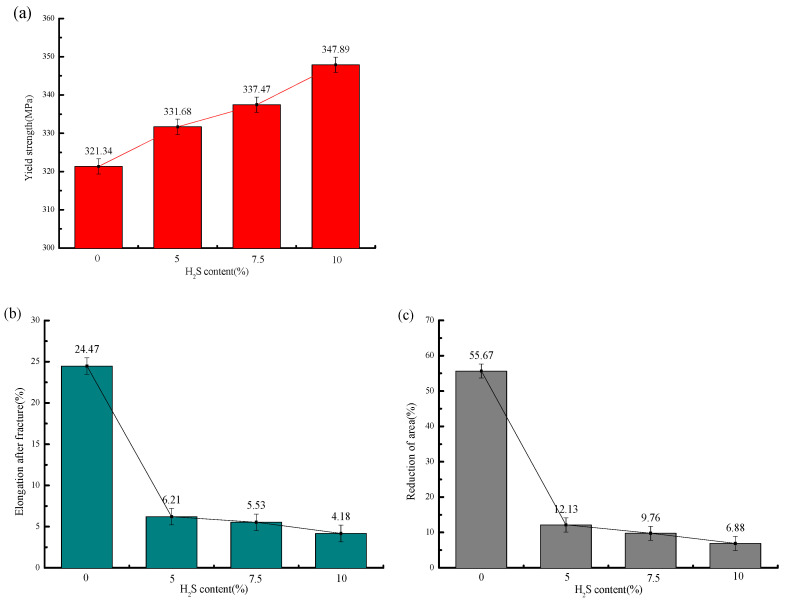
Mechanical properties of 20# steel under different H_2_S concentrations at 30 °C: (**a**) Variation in yield strength with H_2_S concentration; (**b**) variation in post-fracture elongation with H_2_S concentration; (**c**) variation in reduction in area (RA) with H_2_S concentration.

The stress–strain curves and mechanical property parameters at 30 °C with different H_2_S contents are shown in Figure 5 and Figure 6 and Table 6, from which it can be seen that the yield strength of the sample in air is 321.34 MPa, and those under different H_2_S contents are 331.68 MPa, 337.47 MPa, and 347.89 MPa, in turn, increasing by 3.2%, 5.1%, and 8.3%, respectively.

The elongation after fracture in air is 24.47%, while those under different H_2_S contents are 6.21%, 5.53%, and 4.18%, in sequence, decreasing by 18.26%, 18.94%, and 20.29%, respectively. The reduction in area in air is 55.67%, and those under different H_2_S contents are 12.13%, 9.76%, and 6.88%, in sequence, decreasing by 78.2%, 82.5%, and 87.6%, respectively. It can be seen that the variation ranges of the elongation after fracture and reduction in area from air to the working condition with 5% H_2_S are significantly larger than those from the working condition with 5% H_2_S to that with 10% H_2_S.

With increasing H_2_S concentration, the yield strength of 20# steel exhibited a linear increase, while both elongation after fracture and reduction in area progressively decreased. The most dramatic deterioration occurred between the test in air and the test with a 5% H_2_S exposure, where elongation plummeted by 74.6%. Beyond 5% H_2_S, the rate of ductility loss significantly moderated, showing that further increases in H_2_S concentration (5–10%) had diminishing effects on these mechanical properties. This concentration-dependent behavior demonstrates that higher H_2_S levels exacerbate mechanical degradation, substantially increasing the susceptibility of 20# steel to brittle fracture.

#### 3.1.3. Stress Corrosion Susceptibility Assessment

**Figure 7:**
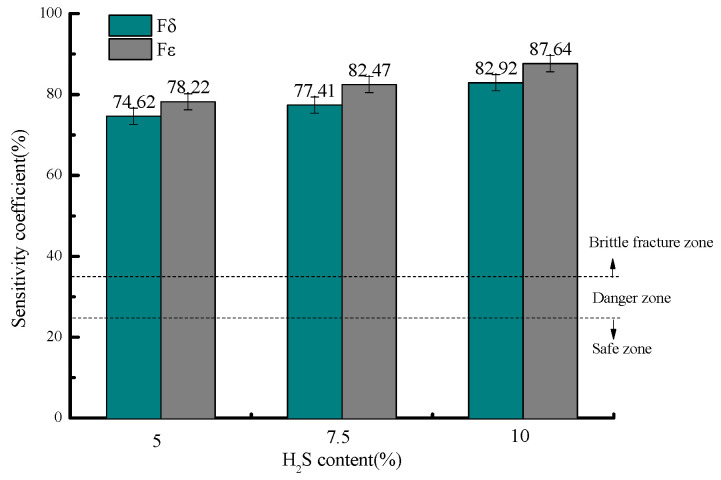
Variation in stress corrosion susceptibility coefficients with H_2_S concentration.

Figure 7 presents the stress corrosion susceptibility coefficients at 30 °C under different H_2_S concentrations, showing elongation sensitivity coefficients (F_δ_) of 74.62%, 77.41%, and 82.92%, and reduction in area sensitivity coefficients (F_ε_) of 78.22%, 82.47%, and 87.64% for the tested H_2_S levels, respectively.

All measured stress corrosion susceptibility coefficients (F_δ_ and F_ε_) under various H_2_S concentrations exceed 35%, placing 20# steel in the brittle fracture zone for stress corrosion cracking (SCC) and indicating an extremely high likelihood of SCC occurrence under these conditions. The risk escalates with increasing H_2_S concentration, reaching maximum susceptibility at 10% H_2_S content.

#### 3.1.4. Microstructural Analysis

**Figure 8:**
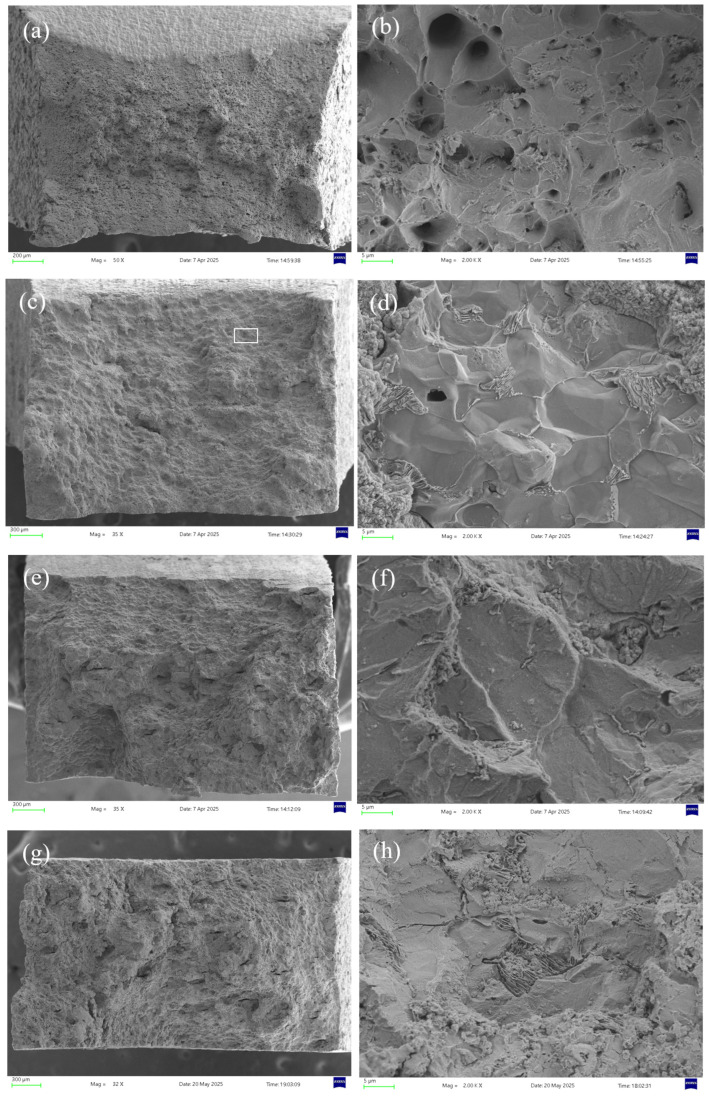
Fracture surface micrographs of 20# steel pipeline under different H_2_S concentrations at 30 °C: (**a**) air, 50× magnification; (**b**) air, 2000× magnification; (**c**) 5% H_2_S, 35× magnification; (**d**) 5% H_2_S, 2000× magnification; (**e**) 7.5% H_2_S, 35× magnification; (**f**) 7.5% H_2_S, 2000× magnification; (**g**) 10% H_2_S, 32× magnification; (**h**) 10% H_2_S, 2000× magnification.

Figure 8 shows the micro-morphologies under different H_2_S concentrations at 30 °C. In air, the specimen exhibits significant necking with numerous fine dimples distributed across the rough fracture surface, indicative of substantial plastic deformation and ductile fracture [28]. In H_2_S/CO_2_ environments, the fracture surface shows corrosion features with markedly reduced necking, where both the quantity and size of dimples decrease progressively with increasing H_2_S concentration. At 10% H_2_S, secondary cracks and inclusions appear on the fracture surface, confirming brittle fracture behavior.

**Figure 9:**
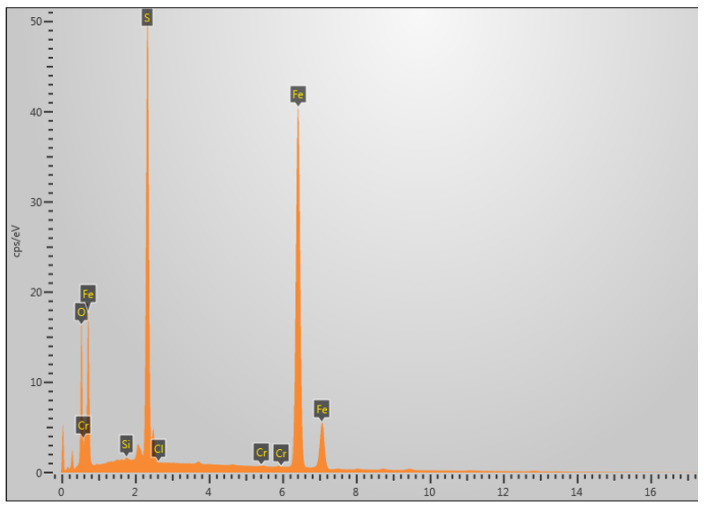
Elemental composition analysis of fracture surface at 30 °C in 5% H_2_S environment.

The area selected for EDS analysis is shown in the white box in Figure 8c. Figure 9 presents the EDS (energy-dispersive X-ray spectroscopy) analysis of the fracture surface under 5% H_2_S and CO_2_ conditions at 30 °C, revealing that the primary elemental composition consists of S, Fe, and O. It is speculated that the main corrosion products are FeO, FeS, etc. [29].

### 3.2. Effect of Temperature

#### 3.2.1. Macro-Morphological Analysis

**Figure 10:**
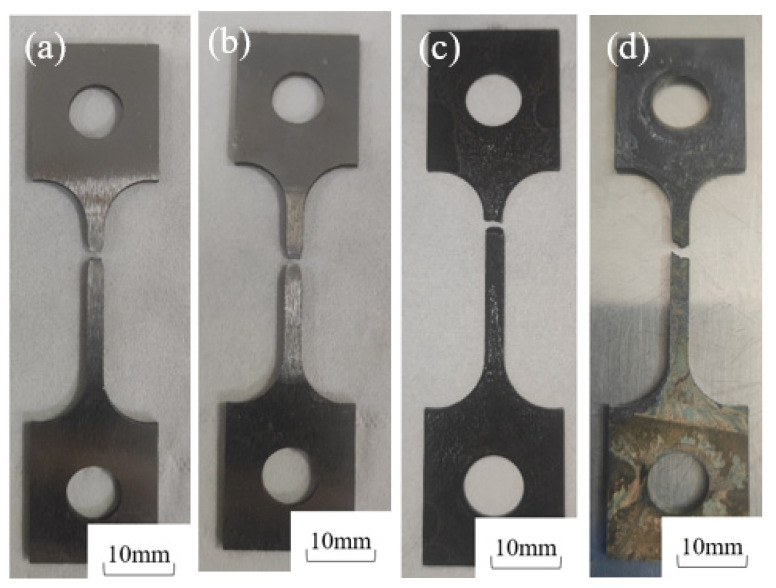
Macro-fracture morphologies of 20# steel at different temperatures: (**a**) 30 °C air; (**b**) 60 °C air; (**c**) 30 °C + 5% H_2_S + 5% CO_2_; (**d**) 60 °C + 5% H_2_S + 5% CO_2_.

Figure 10 compares the post-fracture macro-morphologies in air and in sulfur-containing environments (5% H_2_S) at 30 °C and 60 °C, revealing distinct deformation in air but brittle fractures in H_2_S/CO_2_ conditions. The 30 °C sulfur-exposed specimens exhibited flatter fracture surfaces and darker coloration than those at 60 °C, demonstrating that lower temperatures reduce 20# steel’s resistance to H_2_S stress corrosion while increasing susceptibility. Fracture times dropped drastically from 69 h (air, 30 °C) and 59 h (air, 60 °C) to 17 h and 29 h, respectively, in sulfur-containing environments.

#### 3.2.2. Mechanical Properties Analysis

**Figure 11:**
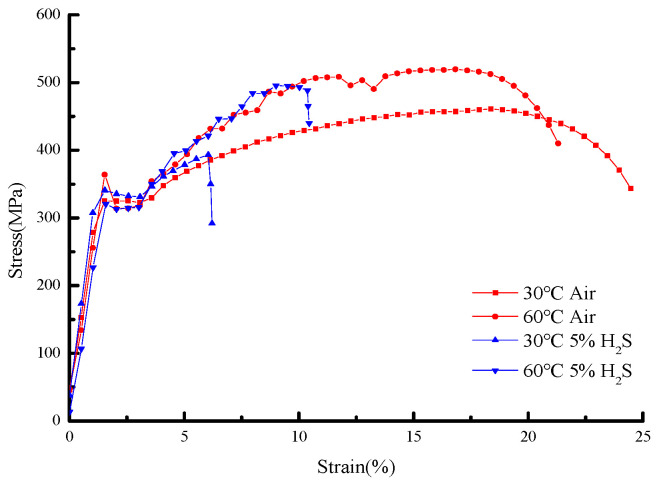
Stress–strain curves of 20# steel under different temperature environments.

**Figure 12:**
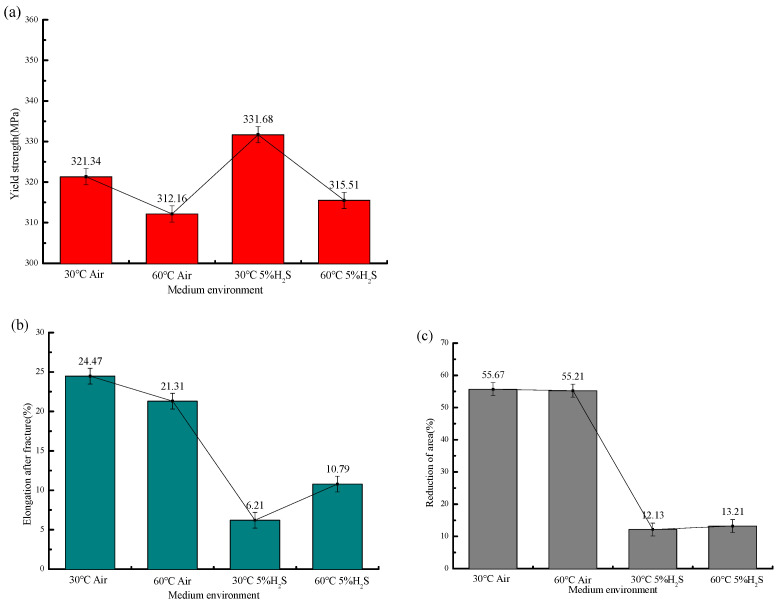
Mechanical properties of 20# steel at different temperatures: (**a**) Variation in yield strength with temperature; (**b**) variation in post-fracture elongation with temperature; (**c**) variation in reduction in area (RA) with temperature.

Figure 11 and Figure 12 present the stress–strain curves and mechanical properties under different temperatures in air and sulfur-containing environments. The data show that in air, the yield strength decreases from 321.34 MPa at 30 °C to 312.16 MPa at 60 °C (a 2.9% reduction), while in the sulfur-containing environment, it decreases from 331.68 MPa to 315.51 MPa (a 4.9% reduction), demonstrating that temperature has an insignificant effect on yield strength.

In air environments, the post-fracture elongation decreased from 24.47% to 21.31% (a 12.9% reduction) and the reduction in area (RA) from 55.67% to 55.21% (a 0.8% reduction) with temperature variation, whereas in sulfur-containing environments, the elongation increased from 6.21% to 10.37% (a 66.9% increase) and RA from 12.13% to 13.21% (an 8.9% increase). This demonstrates that temperature exerts a more pronounced influence on ductility parameters (elongation and RA) in sulfur-containing environments compared to in air. The specific mechanical performance parameters, fracture time, and sensitivity coefficient are shown in Table 7.

These results demonstrate that with increasing temperature, 20# steel exhibits decreased post-fracture elongation in air but increased elongation in H_2_S environments, along with a larger reduction in area and reduced yield strength, indicating weakened resistance to plastic deformation and confirming that elevated temperatures lower the risk of sulfide stress cracking in 20# steel.

#### 3.2.3. Stress Corrosion Susceptibility Evaluation

**Figure 13:**
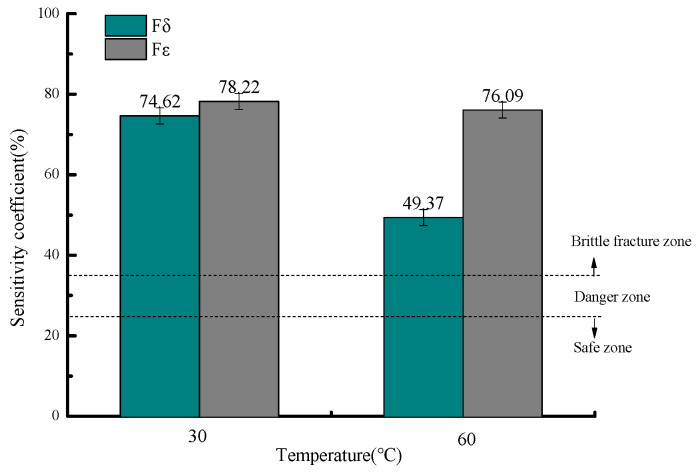
Variation in stress corrosion susceptibility coefficients with temperature.

Figure 13 shows the stress corrosion susceptibility coefficients under different temperatures in both air and sulfur-containing environments, where the elongation sensitivity coefficients (F_δ_) are 74.62% and 49.37%, and the reduction in area sensitivity coefficients (F_ε_) are 78.22% and 76.09%, at the respective temperatures.

The stress corrosion susceptibility coefficients (F_δ_ and F_ε_) at different temperatures all exceed 35%, placing the material in the brittle fracture zone for stress corrosion cracking (SCC) and indicating an extremely high likelihood of SCC occurrence in this environment. Notably, the risk decreases with increasing temperature, with the highest SCC susceptibility observed at 30 °C [30].

#### 3.2.4. Microscopic Morphology Analysis

**Figure 14:**
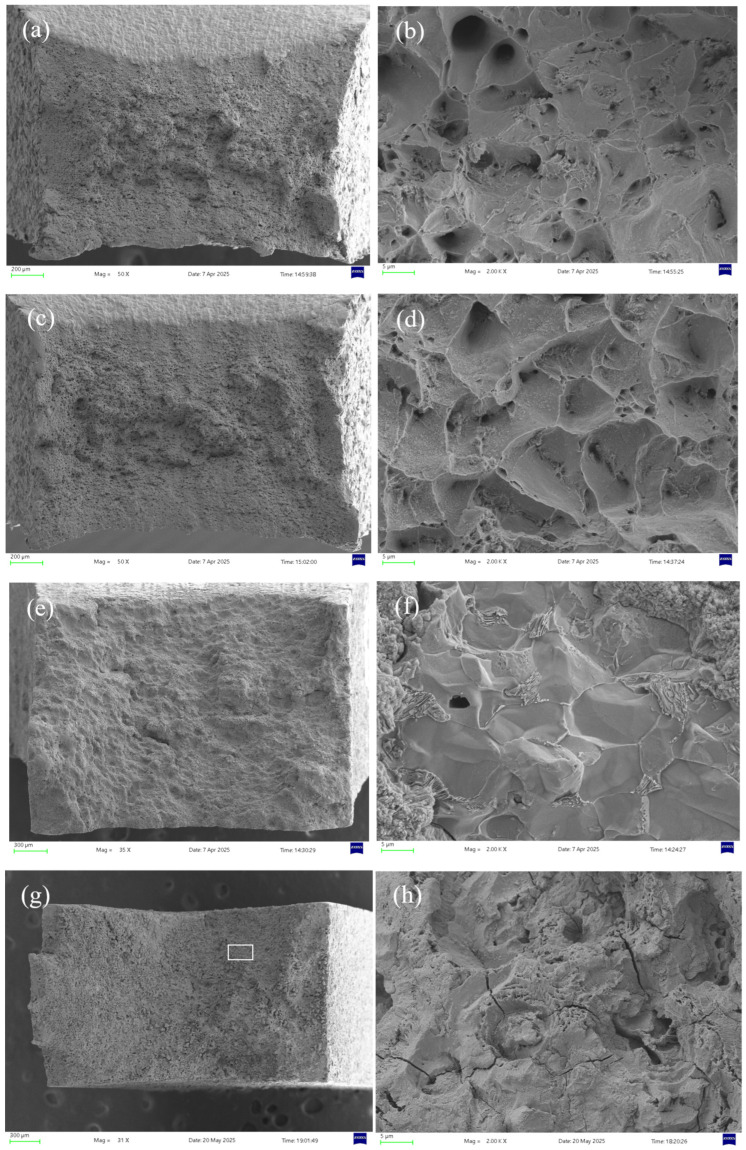
Fracture surface micrographs of 20# steel pipeline at different temperatures: (**a**) 30 °C air, 50× magnification; (**b**) 30 °C air, 2000× magnification; (**c**) 60 °C air, 50× magnification; (**d**) 60 °C air, 2000× magnification; (**e**) 30 °C 5% H_2_S, 35× magnification; (**f**) 30 °C 5% H_2_S, 2000× magnification; (**g**) 60 °C 5% H_2_S, 31× magnification; (**h**) 60 °C 5% H_2_S, 2000× magnification.

Figure 14 compares the microscopic morphologies in air and sulfur-containing environments at different temperatures. In air, the sample at 30 °C exhibits more numerous and larger dimples than at 60 °C, indicating greater ductility. However, in H_2_S-containing environments, both temperatures show brittle fracture characteristics—the 30 °C specimen displays fewer secondary cracks and a smoother fracture surface, while the 60 °C sample contains more secondary cracking. This further confirms higher stress corrosion susceptibility at 30 °C.

**Figure 15:**
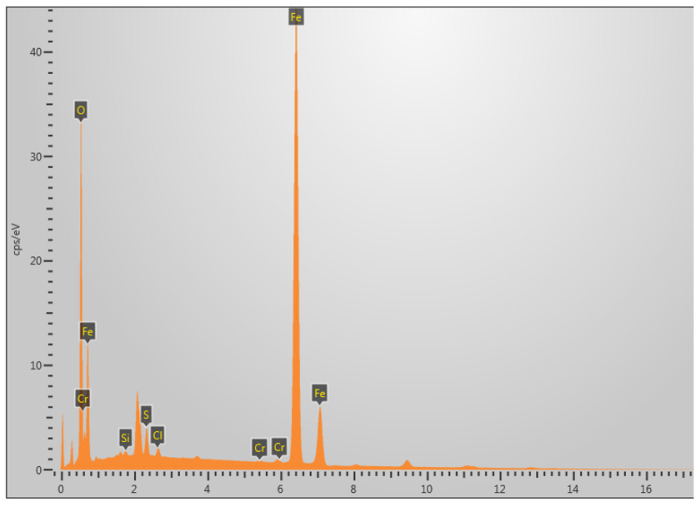
Elemental composition analysis of fracture surface at 60 °C in 5% CO_2_/H_2_S environment.

The area selected for EDS analysis is shown in the white box in Figure 14g. Figure 15 shows the EDS analysis at 60 °C under 5% H_2_S and CO_2_ conditions, revealing that the fracture surface primarily contains the elements S, Fe, and O. It is speculated that the main corrosion products are FeO, FeS, etc. [29].

## 4. Discussion and Analysis

The results of this study indicate that the H_2_S content and temperature have a significant impact on the sensitivity of SSCC in 20# steel welds, which is rooted in their regulatory effects on the dominant mechanism and process of stress corrosion cracking. Based on the observed phenomenon in this experiment—that is, as the concentration of H_2_S increases, the elongation at break and the reduction in cross-sectional area of the material decrease, and the brittleness characteristics intensify (as stated in the first point of Section 5)—it can be inferred that the stress corrosion cracking mechanism dominated by hydrogen embrittlement is the dominant mechanism leading to the deterioration in the performance of the 20# steel weld under the high-sulfur conditions simulated in this experiment.

The stress corrosion cracking process dominated by hydrogen embrittlement in H_2_S environment is shown in Equation (3).

(3) $$2H^{+}+2e^{-}\to H_{2}$$

The HS^−^ and S^2−^ that are dissociated after H_2_S dissolves in water will adsorb on the metal surface, strongly hindering the recombination of hydrogen atoms into H_2_ molecules and promoting the diffusion of hydrogen atoms into the steel interior [31]. The results of this study show that when the concentration of H_2_S increased from 0 to 5%, the elongation at break sharply decreased by 74% (stated in the first point of Section 5). This cliff-like decline confirms that the initial introduction of H_2_S greatly accelerated the hydrogen evolution reaction, producing a large number of hydrogen atoms and infiltrating into the metal. Due to the embrittlement effect of hydrogen, the bonding force between metal atoms was reduced, resulting in a sharp loss of material toughness and a significant increase in stress corrosion sensitivity.

A finding worth further discussion is that the effect of H_2_S concentration is not linear. This experiment clearly reveals that 0–5% is the sensitive critical range of the material, and after exceeding 5%, the toughness degradation effect (elongation at break only decreased by 2.11%) and sensitivity improvement (increased by 12%) caused by the further increase of H_2_S concentration to 10% are significantly slowed down (stated in the third point of Section 5). This phenomenon can be explained by the “surface adsorption saturation” and “corrosion product film effect”. In the low concentration range (0–5%), the surface active sites of the material are sufficient, and the hydrogen adsorption and permeation rates increase almost linearly with the H_2_S concentration, leading to a sharp manifestation of hydrogen damage effects. When the concentration exceeds the critical value, the hydrogen adsorption on the material surface gradually becomes saturated, and the hydrogen permeation rate reaches a stable plateau. Therefore, further increases in H_2_S concentration weaken the “gain” effect of hydrogen embrittlement.

In addition, as pointed out in references [32], dense sulfide corrosion product films such as FeS are more likely to form in high H_2_S concentration environments. This study also observed the coverage of corrosion product films in SEM microstructure analysis. This film, to some extent, hinders the direct contact between the corrosive medium and the substrate, partially offsetting the accelerated corrosion tendency caused by high H_2_S concentration. The coupling of these two effects, namely, hydrogen adsorption saturation and the physical barrier effect of corrosion product film, jointly leads to a decrease in the sensitivity of materials to changes in H_2_S concentration in high-sulfur (H_2_S > 5%) environments. This discovery has important guiding significance for engineering practice: once the concentration of H_2_S in the environment exceeds the critical value, simply pursuing further reduction in the H_2_S concentration will have a low marginal benefit in improving the material’s resistance to SSCC performance, and other factors such as temperature, stress concentration, and material quality should be paid more attention to.

The effect of temperature shows a different pattern from that of H_2_S concentration. The results of this study show that increasing the temperature from 30 °C to 60 °C can reduce the stress corrosion sensitivity of the material by nearly 30% (as stated in the second point of Section 5), indicating that the low-temperature environment significantly exacerbates the risk of SSCC. This is mainly due to two factors: solubility and reaction kinetics. On the one hand, an increase in temperature will accelerate chemical reactions and hydrogen diffusion rates, theoretically exacerbating hydrogen-induced cracking. On the other hand, the solubility of H_2_S gas in aqueous solution decreases with increasing temperature [33]. In this study, the negative effect of solubility dominated. The increase in temperature leads to a decrease in the number of H_2_S molecules actually involved in the corrosion reaction in the solution, weakening the “source power” of the hydrogen evolution reaction and reducing the amount of hydrogen permeation, thereby reducing the sensitivity to hydrogen embrittlement.

Corrosion product film formation mechanism: The increase in temperature affects the morphology and protective properties of corrosion products. As stated in Section 5, at higher temperatures (60 °C), there is a greater tendency to generate dense FeS or FeS_2_ films with better protective properties [34], which can effectively block the invasion and corrosion of hydrogen; at a low temperature of 30 °C, loose and unprotected products such as Fe_9_S_8_ may be generated, providing channels for hydrogen permeation. The phenomenon observed in this study, where the cross-sectional shrinkage rate increases with increasing temperature (as stated in the second point of Section 5), supports the assertion that the protective film plays a positive role.

In summary, the experimental results of this study clearly indicate that for the 20# steel weld, the H_2_S concentration is the “initiating” factor that triggers cracking, especially below the critical concentration where its impact is most severe; And temperature mainly exerts its “inhibitory” or “promoting” effect by adjusting the corrosiveness of the medium and the characteristics of the product film, with a smaller impact than the H_2_S content.

This conclusion has important broader implications: The importance of material quality control: Given that 20# steel is in a sensitive area in a sulfur-containing environment, it is crucial to strictly control the quality of welds and base materials (such as chemical composition, microstructure, and avoiding hard martensitic structures) and eliminate stress concentration points (through optimized design and post-weld heat treatment). These internal factors are often the triggers for hydrogen-induced cracking. Priority of environmental parameter control: In the design and management of operating conditions, keeping the H_2_S concentration below 5% brings the greatest safety benefits. For environments that are already above 5%, more attention should be paid to temperature monitoring and insulation to avoid equipment operating at low temperatures, which is more operationally and economically viable than further desulfurization.

The stress corrosion cracking of metal materials in a wet H_2_S environment is commonly referred to as sulfide stress corrosion. Compared to other media environments, materials in H_2_S environments suffer from more severe stress corrosion failure due to hydrogen embrittlement [35]. This is because HS^-^ and S^2-^ in H_2_S environments hinder the recombination of hydrogen atoms adsorbed on metal surfaces into hydrogen gas, leading to an increasing concentration of hydrogen atoms on the surface and promoting their diffusion into the matrix [36]. As the partial pressure of H_2_S increases, the sensitivity of the material to stress corrosion cracking increases. When the partial pressure of H_2_S is less than 0.3 kPa, stress corrosion cracking will not occur due to H_2_S. However, when the partial pressure of H_2_S exceeds 0.3 kPa, there is a risk of stress corrosion cracking [7,37].

This study revealed the coupling effect of H_2_S concentration and temperature on the SSCC of 20# steel weld through systematic experiments and deepened the understanding of the hydrogen hydrogen-induced cracking mechanism behind the results through analysis. It provides direct data support and a theoretical basis for the safety design of high-sulfur gathering and transportation systems and risk assessment of in-service equipment.

## 5. Conclusions

(1) An increase in H_2_S concentration elevates the yield strength of 20# steel while progressively reducing both elongation after fracture and reduction in area, with higher H_2_S levels exacerbating mechanical degradation and promoting brittle fracture in the material.
(2) Temperature exhibits negligible influence on the yield strength of 20# steel, while its effect on elongation and reduction in area is less pronounced in air than in sulfur-containing environments. With increasing temperature in H_2_S/CO_2_ conditions, the reduction in area increases and the yield strength decreases.
(3) Under all tested H_2_S concentrations and temperatures, the stress corrosion susceptibility coefficients (F_δ_, F_ε_) of 20# steel exceeded 35%, placing the material in the stress corrosion brittle fracture sensitive zone, with susceptibility increasing with higher H_2_S content or lower temperatures. While elongation and reduction in area showed dramatic decreases when transitioning from air to a 5% H_2_S environment (74% and 78% reductions, respectively), further H_2_S increases from 5% to 10% resulted in progressively diminishing effects, indicating that beyond 5% H_2_S, concentration variations no longer significantly impact stress corrosion susceptibility.

## Figures and Tables

**Table 1:** Chemical composition testing results (wt.%).

Category	C	Si	Mn	P	S	Ni	V	Cr	Ti	Cu	Mo
Base metal	0.144	0.21	0.538	0.011	0.005	0.282	0.002	0.171	0.002	0.368	0.039
Weld metal	0.080	0.183	0.558	0.015	0.016	0.084	0.004	0.050	0.023	0.142	0.013

**Table 2:** Hardness values.

Testing Area	Measured Hardness (HV10)
Base Metal	152,158,149,165
Heat-Affected Zone	214,205,208,183
Weld	163,170,153,166

**Table 3:** Mechanical performance parameters of base metal.

Mechanical Properties	Tensile Strength Rm/MPa	Yield Strength Rel/MPa	Post-Fracture Elongation Rate A/%
Base material	465	303	20.5

**Table 4:** pH values as a function of experimental condition.

Temperature	H_2_S Molar Content	pH
30 °C	5%	3.56
	7.5%	3.53
	10%	3.5
60 °C	5%	3.56
	7.5%	3.51
	10%	3.48

**Table 5:** Chemical composition of simulated field solution.

Component	CO_3_^2−^	HCO^3−^	SO_4_^2−^	K^+^	Ca^2+^	Mg^2+^	Cl^−^
Content	5 ppm	90 ppm	10 ppm	5 ppm	250 ppm	150 ppm	50,000 ppm

**Table 6:** Mechanical properties under different H_2_S concentrations.

Medium Environment	Yield Strength/MPa	Elongation After Fracture/%	Reduction in Area/%	Fracture Time/h	F_δ_/%	F_ε_/%
30 °C + air	321.34	24.47	55.67	69	-	-
30 °C + 5% H_2_S + 5% CO_2_	331.68	6.21	12.13	17	74.62	78.22
30 °C + 7.5% H_2_S + 5% CO_2_	337.47	5.53	9.76	15	77.41	82.47
30 °C + 10% H_2_S + 5% CO_2_	347.89	4.18	6.88	12	82.92	87.64

**Table 7:** Mechanical properties at different temperatures.

Medium Environment	Yield Strength/MPa	Elongation After Fracture/%	Reduction in Area/%	Fracture Time/h	F_δ_/%	F_ε_/%
30 °C + air	321.34	24.47	55.67	69	-	-
60 °C + air	312.16	21.31	55.21	59	-	-
30 °C + 5% H_2_S + 5% CO_2_	331.68	6.21	12.13	17	74.62	78.22
60 °C + 5% H_2_S + 5% CO_2_	315.51	10.37	13.21	29	49.37	76.09

## Data Availability

Data are contained within the article.
